# *Hovenia dulcis* Thunb. monofloral honey attenuates LPS-induced inflammation and endotoxemia through the activation of the Nrf2/HO-1 axis^☆^

**DOI:** 10.1016/j.jtcme.2024.09.003

**Published:** 2024-09-17

**Authors:** Wisurumuni Arachchilage Hasitha Maduranga Karunarathne, Sungjoon Na, Mi-Hwa Lee, Chang-Hee Kang, Yung Hyun Choi, Gi-Young Kim

**Affiliations:** a Department of Marine Life Science, Jeju National University, Jeju, 63243, Republic of Korea; b Department of Biosystems Technology, Faculty of Technology, University of Ruhuna, Matara, 81000, Sri Lanka; c Forest Bioresources Department, National Institute of Forest Science, Suwon, 16631, Republic of Korea; d Nakdonggang National Institute of Biological Resources, Sangju, 37242, Republic of Korea; e Department of Biochemistry, College of Korean Medicine, Dong-Eui University, Busan, 47227, Republic of Korea

**Keywords:** *Hovenia dulcis* thunb., Monofloral honey, Inflammation, Endotoxemia, Antioxidant

## Abstract

Monofloral honey derived from *Hovenia dulcis* Thunb*.* (HMH) is known for its antimicrobial and antioxidant properties. However, its potential to alleviate the inflammatory response has not yet been explored. The aim of this study was to investigate the anti-inflammatory and anti-endotoxemic effects of HMH. The findings showed that HMH did not exhibit toxicity to RAW 264.7 macrophages at low concentrations and suppressed the production of proinflammatory mediators, such as nitric oxide and prostaglandin E_2_, as well as cytokines, including tumor necrosis factor-α and interleukin-12 in lipopolysaccharide (LPS)-stimulated RAW 264.7 macrophages, by inhibiting NF-κB activation. Additionally, HMH prevented mortality and abnormalities in LPS-microinjected zebrafish larvae along with the inhibition of proinflammatory genes. In addition, HMH was found to reduce mitochondrial membrane potential depolarization and mitochondrial reactive oxygen species production in both LPS-stimulated RAW 264.7 macrophages and zebrafish larvae. Furthermore, HMH induced the expression of nuclear factor erythroid 2-related factor 2 (Nrf2) and heme oxygenase-1 (HO-1) and promotes the nuclear translocation of Nrf2. The anti-inflammatory effects of HMH are mediated via the Nrf2-HO-1 axis, and an HO-1 inhibitor reverses HMH-induced responses. This study is the first to demonstrate the anti-inflammatory and anti-endotoxemic effects of HMH, highlighting its potential as a therapeutic.

## Graphical abstract

**Figure:**
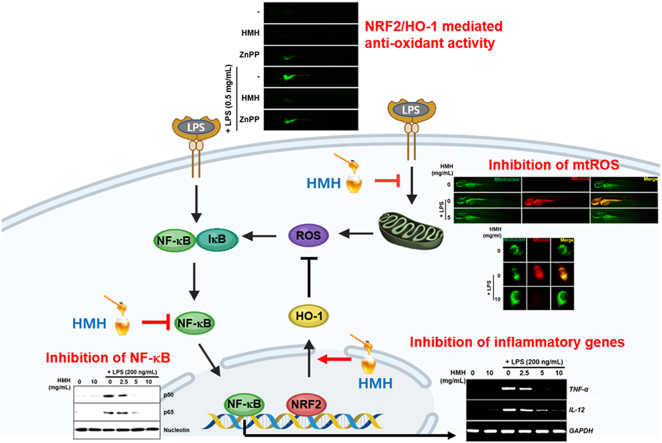

## 1 Introduction

Endotoxemia, caused by the immune response to lipopolysaccharide (LPS) in gram-negative bacteria is a severe condition that can lead to life-threatening sepsis due to the excessive release of proinflammatory cytokines into the bloodstream.^1^^,^^2^ It is a significant cause of mortality in intensive care units and is characterized by hyperinflammation, fever, rapid heartbeat, low blood pressure, and organ dysfunction.^3^ In endotoxemia, the release of inflammatory cytokines is deregulated, leading to a cytokine storm, resulting in the unrestricted release of cytokines.^4^ The initial phase of endotoxemia involves the polarization of M1-like macrophages and the massive release of inflammatory cytokines, such as tumor necrosis factor-α (TNF-α), interleukine-12 (IL-12), and IL-6, as well as free radicals, such as reactive oxygen species (ROS) and reactive nitrogen species (RNS).^5^^,^^6^ These responses can cause severe inflammation and organ damage.^7^ In contrast, the polarization of macrophages toward the M2-like phenotype leads to the production of anti-inflammatory cytokines such as IL-10, transforming growth factor-β, and arginase-1, which help suppress excessive inflammation and promote tissue repair.^5^ The shift from M1-like to M2-like phenotype is a critical step in resolving inflammation and preventing the development of endotoxemia. However, in some cases, this shift may be incomplete or impeded, leading to the development of a persistent inflammatory state, contributing to the progression of endotoxemia.^8^ Thus, the inhibition of M1-like macrophages is a crucial target for the development of therapies to treat endotoxemia.

The excessive and prolonged production of free radicals, such as ROS and RNS, has been implicated in the pathogenesis of sepsis by causing mitochondrial damage.^9^ In endotoxemia, the expression of antioxidant enzymes, such as superoxide dismutase and glutathione peroxidase, is downregulated in sinusoidal phagocytes, leading to increased levels of ROS and subsequent hepatic damage.^10^ The overexpression of glutathione peroxidase has been found to improve the survival rate and blood pressure, as well as downregulate proinflammatory mediators, such as nitric oxide (NO) and TNF-α, in mice treated with high doses of LPS.^11^ Sustainable nanosheet antioxidants have also been demonstrated to attenuate ROS and RNS production in mice with sepsis, inhibit proinflammatory cytokines, and improve mortality.^12^ Nuclear factor erythroid 2-related factor (Nrf2) has been identified as a key regulator of the expression of antioxidant proteins, including heme oxygenase-1 (HO-1), which protects against endotoxemia.^13^^,^^14^ The induction of HO-1 has been shown to reduce mortality and inflammatory responses in non-surgical preterm endotoxemia.^15^ In recent decades, standard antioxidant therapy has been proposed as a potential therapy for reducing the severity and mortality of endotoxemia.^16^^,^^17^

Honey is a natural substance produced by bees and has been valued for its nutritional and health-promoting properties for centuries.^18^^,^^19^ It contains a variety of sugars, minerals, vitamins, and plant-derived phenolic compounds that have anti-inflammatory, antioxidant, and antimicrobial properties.^20^ However, the composition of honey can vary depending on several factors, making it essential to study monofloral honey to understand the medicinal effects of honey from single flower sources. Recent studies have highlighted the potential therapeutic effects of *Hovenia dulcis* Thunb*.* monofloral honey (HMH), supplied by the National Institute of Forest Science (Suwon, Gyeonggi-do, Republic of Korea), in regulating antimicrobial and antioxidant activities, with its comparable levels of total phenolic content compared to Acacia honey.^21^^,^^22^ However, the anti-endotoxemia mechanisms of HMH and its potential therapeutic effects on the inflammatory response and oxidative stress require further evaluation. Therefore, this study investigated the role of HMH in regulating the inflammatory response and oxidative stress associated with endotoxemia and its potential therapeutic effects.

## 2 Material and methods

### 2.1 HMH preparation

Twenty-six Hovenia trees and honeybees (*Apis* *cerana* Fabricius) were cultivated in a net house (23 × 13 × 9 m; W × D × H) at the National Institute of Forest Science to obtain HMH. The honey was harvested in July 2021 and stored at 4 °C in a refrigerator until use.

### 2.2 Reagents and antibodies

Dulbecco's modified Eagle's medium (DMEM), fetal bovine serum (FBS), and antibiotic mixture were purchased from WELGENE (Daegu, Republic of Korea). LPS from *Escherichia coli* O55:B5, 3-(4,5-dimethylthiazol-2-yl)-2,5-diphenyl-tetrazolium bromide (MTT), and 4′6-diamidine-2′-phenylindole dihydrochloride (DAPI) were purchased from Sigma-Aldrich (St. Louis, MO, USA). 2′,7′-Dichlorodihydrofluorescein diacetate (DCFDA) and 4-amino-5-methylamino-2′7′-dichlorodihydrofluorescein diacetate (DAF-FMDA) were purchased from Molecular Probes (Eugene, OR, USA). Antibodies against inducible nitric oxide synthase (iNOS, sc-7271, 1:000), cyclooxygenase-2 (COX-2, sc-19999, 1:1000), p50 (sc-8414, 1:250), p65 (sc-8008, 1:250), Nrf2 (sc-365949, 1:1000), HO-1 (sc-10789, 1:1000), β-actin (sc-69879, 1:1000), and nucleolin (sc-13057, 1:250) were purchased from Santa Cruz Biotechnology (Dallas, Texas, USA). Alexa Fluor 488-conjugated secondary antibodies were purchased from Abcam (Cambridge, UK). Dako Faramount Aqueous Mounting Medium was obtained from Dako (Carpinteria, CA, USA). Peroxidase-labeled anti-rabbit and anti-mouse immunoglobulins were purchased from KOMA BIOTECH (Seoul, Republic of Korea). All other chemicals were obtained from Sigma-Aldrich.

### 2.3 Cell culture and MTT assay

RAW 264.7 macrophages were obtained from American Type Culture Collection (Manassas, VA, USA) and cultured in DMEM supplemented with 5 % FBS and antibiotic mixtures at 37 °C in a stable environment with 5 % CO_2_. The viability of RAW 264.7 macrophages after treatment with different concentrations of HMH (0–100 mg/mL) was evaluated using the MTT assay. Briefly, cells were seeded in 24-well plates (0.5 mL) at a density of 1 × 10^5^ cells/mL for 12 h and then treated with HMH for 24 h. After treatment, MTT solution (0.5 mg/mL) was added to the cells and incubated for 30 min. The medium was then removed and dimethyl sulfoxide was added to dissolve the dark formazan crystals. Absorbance was measured at 570 nm using a microplate spectrophotometer (BioTek Instruments, Winooski, VT, USA). In parallel, cell morphology was observed using a stereomicroscope (Macrotech, Goyang, Gyeonggido, Republic of Korea).

### 2.4 Analysis of cell viability and total cell count using flow cytometry

RAW 264.7 macrophages were seeded at a density of 1 × 10^5^ cell/mL and treated with HMH (0–100 mg/mL) for 24 h. Cells were collected, washed with ice-cold phosphate-buffered saline (PBS), and incubated with a Muse Cell Count & Viability Kit (Luminex, Austin, TX, USA) for 5 min. The cell viability and total cell count were analyzed using a Muse Cell Analyzer (Luminex).

### 2.5 NO production and staining

RAW 264.7 macrophages were seeded at a density of 1 × 10^5^ cells/mL in 24-well plates and pre-treated with HMH (0–10 mg/mL) for 2 h, followed by stimulation with 200 ng/mL LPS for 24 h to induce NO production. The level of NO in the culture supernatant was determined using the Griess reagent assay. Equal volumes of cell supernatant and Griess reagent (1 % sulfanilamide in 5 % phosphoric acid and 0.1 % naphthylethylenediamine dihydrochloride) were mixed and incubated for 15 min. Absorbance was measured at 540 nm using a microplate spectrophotometer (BioTek Instruments). The nitrite concentration was quantified by comparison with a standard sodium nitrite curve. In a parallel experiment, intracellular NO production was assessed using an NO indicator, DAF-FMDA. RAW 264.7 macrophages were incubated with 1 μM DAF-FMDA for 30 min at 37 °C, and the fluorescence was visualized using a CELENA S Digital Imaging System (Logos Biosystems, Anyang, Gyeonggi-do, Republic of Korea).

### 2.6 Isolation of RNA and reverse transcription polymerase chain reaction (RT-PCR)

Total RNA was isolated from cells using the Easy-BLUE Total RNA Extraction Kit (iNtRON Biotechnology, Seongnam, Republic of Korea), and its concentration was quantified at 260 nm using a NanoDrop spectrophotometer (Eppendorf, Hamburg, Germany). cDNA was synthesized from 2 μg of total RNA using MMLV reverse transcriptase (Bioneer, Daejeon, Republic of Korea). PCR was performed using EzWay PCR Master Mix (KOMA BIOTECH, Seoul, Republic of Korea). The gene-specific primers used are listed in Table 1. The PCR conditions comprised of 27 cycles of denaturation at 95 °C for 45s, annealing at the indicated Tm (Table 1) for 45 s, and extension at 72 °C for 1 min. The relative expression of all genes was normalized to glyceraldehyde-3-phosphate dehydrogenase (*GAPDH*) for mice and β-actin for zebrafish.

**Table 1:** Primer sequences used in this study for RT-PCR.

Species	Gene	Primer sequence (5′–3′)	Size (bp)	Tm (°C)
mouse	*TNF-α*	F: 5′-ATGAGCACAGAAAGCATGAT-3′ R: 5′ -TACAGGCTTGTCACTCGAAT-3′	276	53
	*IL-12*	F: 5′-AAGACATCACACGGGACCAA-3′ R: 5′-GAGGATACCACTTCCCAACAG-3′	313	63
	*iNOS*	F: 5′-CCTCCTCCACCCTACCAAGT-3′ R: 5′-CACCCAAAGTGCTTCAGTCA-3′	199	55
	*COX-2*	F: 5′-TGCTGTACCAGCAGTGGCAA-3′ R: 5′-GCAGCCATTTCCTTCTCTCC-3′	141	58
	*Nrf2*	F: 5′-TGGACGGGACTATTGAAGGC-3′ R: 5′-GCCGCCTTTTCAGTAGATGG-3′	735	59
	*HO-1*	F: 5′-TGAAGGAGGCCACCAAGGAG-3′ R: 5′ -AGAGGTCACCCAGGTAGCGG-3′	375	61
	*GAPDH*	F: 5′-ACCACAGTCCATGCCATCAC-3′ R: 5′-CACCACCCTGTTGCTGTAGC-3′	480	63
zebrafish	*iNOS*	F: 5′-GGAGATGCAAGGTCAGCTTC-3′ R: 5′-GGCAAAGCTCAGTGACTTCC-3′	137	58
	*COX-2a*	F: 5′-CCTGTTGTCAAGGTCCCATT-3′ R: 5′-TCAGGGATGAACTGCTTCCT-3′	201	57
	*TNF-α*	F: 5′-TAGAACAACCCAGCAAAC-3′ R: 5′-ACCAGCGGTAAAGGCAAC-3′	149	57
	*β-actin*	F: 5′-CGAGCGTGGCTACAGCTTCA-3′ R: 5′-GACCGTCAGGCAGCTCATAG-3′	155	61

*iNOS*; inducible nitric oxide synthase, *COX-2*; cyclooxygenase-2, *TNF-α*; tumor necrosis factor-α, *IL-12*; interleukin-12, *Nrf2*; nuclear factor erythroid 2-related factor 2, *HO-1*; heme oxygenase-1, and *GAPDH*; glyceraldehyde 3-phosphate dehydrogenase.

bp, base pair; Tm, melting temperature; F, forward; R, reverse.

### 2.7 Western blot analysis

Total cellular proteins were extracted using RIPA Lysis Buffer (iNtRON Biotechnology) supplemented with a protease inhibitor cocktail (Sigma-Aldrich). Cytoplasmic and nuclear extracts were prepared using NE-PER Nuclear and Cytosolic Extraction Reagents (Pierce, Rockford, IL, USA). The protein concentrations were determined using the Bio-Rad Protein Assay Kit (Bio-Rad, Hercules, CA, USA). Briefly, the RIPA lysis buffer was applied to the cells on ice for 30 min, and lysates were then centrifuged at 14,000×*g* for 10 min at 4 °C. The extracts were either immediately used for western blotting or stored at −80 °C. Proteins were separated using SDS-polyacrylamide gel electrophoresis, transferred to nitrocellulose membranes (Amersham, Arlington Heights, IL, USA), and immunoblotted using specific primary antibodies for 12 h. The membranes were treated with HRP-conjugated anti-mouse or anti-rabbit immunoglobulin for 2 h. The superSignal West Femto Maximum Sensitivity Substrate (Thermo Fisher Scientific, Waltham, MA, USA) was used to visualize the protein bands, and a ChemiDoc Touch Imaging System (GE Healthcare Bio-Sciences AB, Uppsala, Sweden) was used to detect protein expression levels. Relative expression was normalized to the expression levels of β-actin and nucleolin.

### 2.8 Enzyme immunosorbent assay (ELISA)

RAW 264.7 macrophages were treated with HMH (0–10 mg/mL) for 2 h followed by LPS (200 ng/mL) stimulation for 24 h. The concentrations of PGE_2_ (Cayman Chemicals, Ann Arbor, MI, USA), TNF-α (PeproTech, Cranbury, NJ, USA), and IL-12 (PeproTech) in the culture supernatant were measured using ELISA according to the manufacturer's instructions.

### 2.9 Immunofluorescence analysis

To evaluate the localization of p65, RAW 264.7 macrophages were fixed with 4 % paraformaldehyde for 15 min, permeabilized with 0.1 % Triton X-100 for 5 min, and then blocked with 10 % donkey serum for 1 h. The cells were then incubated with anti-p65 primary antibody (1:100 in 10 % donkey serum) for 16 h at 4 °C and subsequently incubated with Alexa Fluor 488-conjugated secondary anti-mouse antibody for 1 h at room temperature. Nuclei were counterstained with DAPI for 10 min. The cells were washed with PBS, mounted, and analyzed using the CELENA S Digital Imaging System (Logos Biosystems).

### 2.10 Luciferase reporter assay

To evaluate the activity of NF-κB, RAW 264.7 macrophages were transfected with 1 μg of NF-κB-Luc and 0.1 μg of *Renilla* plasmid using StremGENE9 Transfection Reagents (Promega, Carlsbad, CA, USA). After 48 h, the culture medium was replaced with fresh DMEM containing 5 % FBS and then treated with HMH (0–10 mg/mL) for 2 h, followed by LPS (200 ng/mL) stimulation for 30 min. Luciferase activity was measured using a Dual-Luciferase Reporter Assay Kit (Promega), and normalized to *Renilla* luciferase activity. The luminescence was measured using a GLOMAX luminometer (Promega).

### 2.11 Mitochondrial membrane potential (ΔΨm) and mitochondrial ROS (mtROS)

To determine ΔΨm, RAW 264.7 macrophages were seeded at a density of 1 × 10^5^ cells/mL and treated with HMH (0–10 mg/mL) for 2 h prior to LPS (200 ng/mL) stimulation for 24 h. The cells were then incubated with the Muse Mitopotential Kit (Luminex), and ΔΨm was measured using the Muse Cell Analyzer (Luminex). In parallel experiments, cells were stained with 5 μM MitoTracker Green for 30 min and subsequently with 10 μM MitoSOX Red in the presence or absence of MitoTEMPO for 10 min. The resulting images were captured using a CELENA S Digital Imaging System (Logos Biosystems).

### 2.12 Zebrafish maintenance

All zebrafish experiments were conducted in accordance with the ARRIVE guidelines^23^ and approved by the Institutional Animal Care and Use Committee (IACUC) of Jeju National University (Jeju Special Self-Governing Province, Republic of Korea) (approval no. 2022-0083). Adult AB strain zebrafish were obtained from the Nakdonggang National Institute of Biological Resources (Sangju, Gyeongsangbukdo, Republic of Korea) and maintained at 28.0 °C with a photoperiod of 14 h of light and 10 h of darkness. To induce natural spawning, the light was turned on in the morning. Embryos were cultured in E3 embryo medium (34.8 g NaCl, 1.6 g KCl, 5.8 g CaCl_2_·2H_2_O, and 9.78 g MgCl_2_·6H_2_O in 1 L of double-distilled water, pH 7.2) supplemented with 1 % methylene blue at 28 °C.

### 2.13 LPS microinjection into zebrafish larvae and toxicity evaluation

Zebrafish embryos at 1 day post-fertilization (dpf) were pretreated with 0.003 % 1-phenyl-2-thiourea (PTU). Subsequently, the zebrafish larvae were incubated with HMH (0–20 mg/mL) for 48 h at 3 dpf. In parallel, zebrafish larvae at 3 dpf were anesthetized, and either 2 nL of LPS (0.5 mg/mL) or PBS was microinjected into the yolk sac using the Drummond NANOJECT III injector (Drummond Scientific, Broomall, PA, USA). The larvae were immediately treated with HMH (0–5 mg/mL) for 48 h in fresh E3 medium. The morphological abnormalities and survival rates were assessed.

### 2.14 Isolation of total zebrafish RNA and RT-PCR

To evaluate the expression *iNOS* and *COX-2a*, total RNA was isolated from zebrafish larvae injected with LPS (0.5 mg/mL, 2 nL) and subsequently treated with HMH for 18 h. Total RNA was extracted using an Easy-BLUE Total RNA Extraction Kit (iNtRON Biotechnology), and cDNA was synthesized using MMLV reverse transcriptase (Bioneer). Specific primers (Table 1) were used to amplify the cDNA using the following PCR conditions: denaturation at 95 °C for 45 s, annealing at the indicated Tm (Table 1) for 45 s, and extension at 72 °C for 1 min. β-Actin was used as a loading control to evaluate the relative expression of *iNOS* and *COX-2a.*

### 2.15 NO staining in zebrafish larvae

To visualize NO production in zebrafish larvae, we used DAF-FMDA. LPS-microinjected zebrafish larvae were treated with HMH (0–5 mg/mL) for 18 h and then incubated with 5 μM DAF-FMDA for 30 min. Fluorescence was visualized using the CELENA S Digital Imaging System (Logos Biosystems).

### 2.16 mtROS staining in zebrafish larvae

To evaluate mtROS production in the zebrafish larvae, staining was performed using MitoSOX Red at a concentration of 10 μM for 30 min with or without the addition of 10 μM MitoTEMPO. The larvae were then treated with 5 μM MitoTracker Green for 10 min in E3 embryo medium. Fluorescent images were captured using a CELENA S Digital Imaging System (Logos Biosystems).

### 2.17 Statistical analysis

The data obtained from RT-PCR and western blotting were visualized using a ChemiDoc Touch Imaging System (GE Healthcare) and quantified using ImageJ software (National Institute of Health, Bethesda, MD, USA). The results represent the mean of at least three independent experiments and are expressed as the standard error of the median (SEM). Statistical analysis was performed using Sigma Plot 12.0 (Systat Software Inc., San Jose, CA, USA) with student's t-test and unpaired one-way analysis of variance with Bonferroni correction.

## 3 Results

### 3.1 Low concentrations of HMH do not exhibit cytotoxicity on RAW 264.7 macrophages

**Fig. 1:**
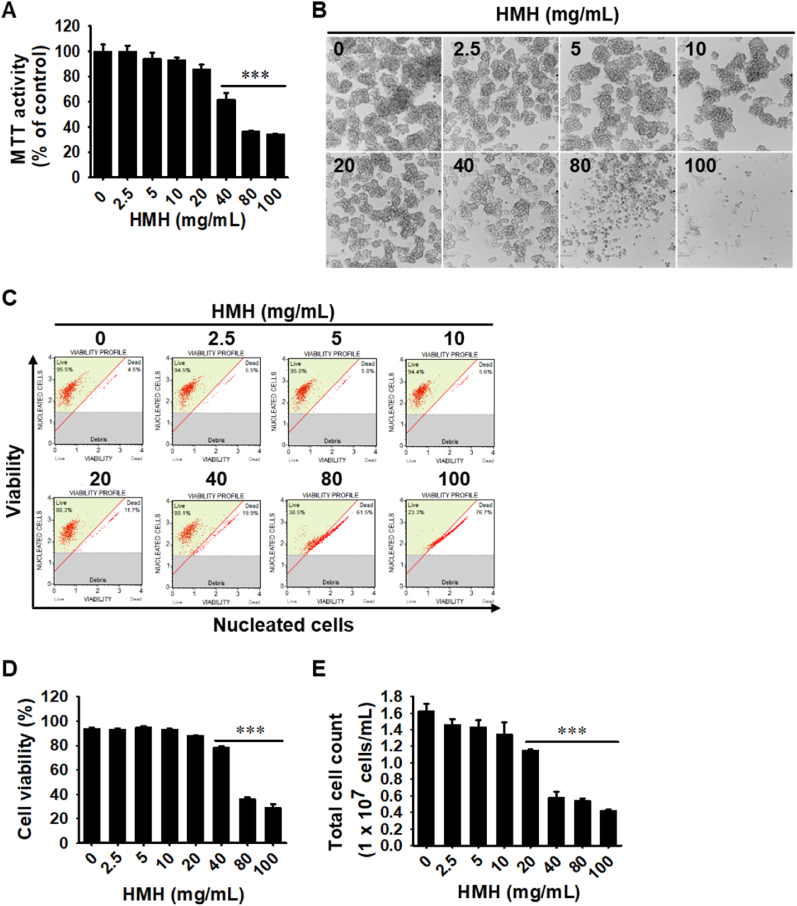
Low concentrations of HMH have no cytotoxicity. RAW 264.7 macrophages (1 × 10^5^ cells/mL) were treated with 0–100 mg/mL HMH for 24 h. (A) The relative cell viability was measured using the MTT assay and compared to the untreated cells. (B) Representative images of the cells were captured using phase-contrast microscopy (× 20). Scale bar = 20 μm. (C) In a parallel experiment using flow cytometry, cell viability (D) and total cell count (E) were measured using a Muse Count & Viability Kit. ∗∗∗*p* < 0.001 vs. untreated cells.

To evaluate the impact of HMH on RAW 264.7 macrophages, we conducted MTT assays, analyzed morphological changes, and performed flow cytometry analysis. After 24-h of exposure to HMH, no reduction in relative cell viability was observed at concentrations up to 20 mg/mL (100 ± 4.6, 94.2 ± 4.6, 93.2 ± 1.7, and 85.7 ± 3.8 at 2.5, 5, 10, and 20 mg/mL HMH; Fig. 1A) compared to untreated cells (100 ± 5.3). Conversely, concentrations of 40 mg/mL or higher significantly reduced relative cell viability (61.7 ± 5.4, 36.6 ± 0.5, and 34.3 ± 0.3 at 40, 80, and 100 mg/mL HMH). We also observed no significant changes in cell morphology at HMH concentrations of 20 mg/mL, whereas high concentrations of HMH (≥40 mg/mL) caused cell debris, floating cells, and contracted cells (Fig. 1B). To confirm whether HMH affected the cell viability and total cell count under the same experimental conditions, we conducted flow cytometry (Fig. 1C). Consistent with the MTT assay results, HMH concentrations up to 20 mg/mL did not reduce cell viability (93.2 ± 0.9, 95.1 ± 0.5, 93.2 ± 0.6, and 87.9 ± 0.2 at 2.5, 5, 10, and 20 mg/mL HMH, respectively) compared to untreated cells (93.5 ± 1.3; Fig. 1D). Concentrations of HMH up to 10 mg/mL did not decrease the total cell number ((1.5 ± 0.1) × 10^7^, (1.4 ± 0.1) × 10^7^, and (1.3 ± 0.2) × 10^7^ cells/mL at 2.5, 5, and 10 mg/mL HMH, respectively), while concentrations of HMH 20 mg/mL or higher significantly decreased the total cell population of viable cells ((1.2 ± 0.3) × 10^7^, (0.6 ± 0.1) × 10^7^, (0.5 ± 0.2) × 10^7^, and (0.4 ± 0.3) × 10^7^ cells/mL at 20, 40, 80, and 100 mg/mL HMH; Fig. 1E). These finding suggest that low concentrations (≤10 mg/mL) of HMH do not exhibit cytotoxic toward RAW264.7 macrophages. Thus, these concentrations were used in subsequent experiments.

### 3.2 HMH inhibits the LPS-induced production of NO and PGE_2_ production in RAW 264.7 macrophages

**Fig. 2:**
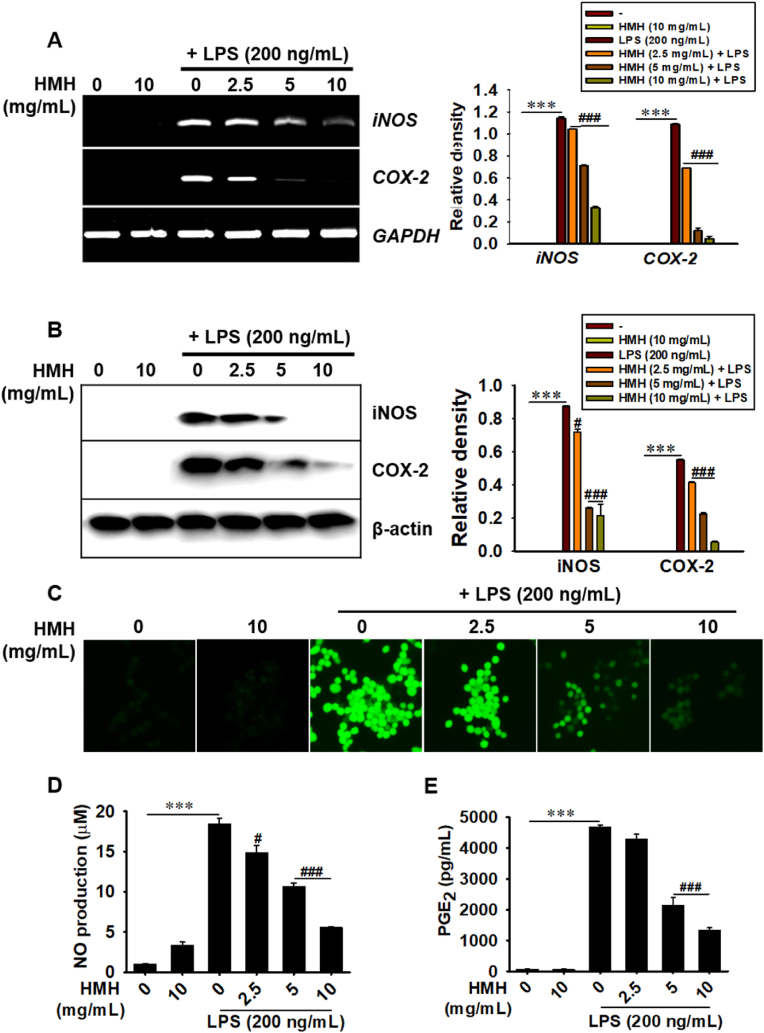
HMH inhibits LPS-induced NO and PGE_2_ production. RAW 264.7 macrophages (1 × 10^5^ cells/mL) were treated with HMH (0–10 mg/mL) for 2 h prior to LPS (200 ng/mL) treatment. (A) Total RNA was extracted 6 h after LPS treatment, and RT-PCR was performed to evaluate the expression of *iNOS* and *COX-2*. *iNOS* and *COX*-2 expression was normalized to *GAPDH* expression. (B) In a parallel experiment, total proteins were extracted at 12 h, and western blotting was performed to evaluate the expression of iNOS and COX-2. iNOS and COX-2 expression was normalized to β-actin expression. (C) Cells were stained using 1 μM DAF-FMDA, and the images were captured using a CELENA S Digital Imaging System. Cell-free culture media were collected 24 h after LPS treatment. (D) Griess reagent assay was performed to quantify NO production, and (E) PGE_2_ levels were quantified using an ELISA assay. ∗∗∗*p* < 0.001 vs. untreated cells; ^#^*p* < 0.05 and ^###^*p* < 0.001 vs LPS-treated cells.

To evaluate the potential anti-inflammatory effect of HMH on LPS-treated RAW 264.7 macrophages, the cells were treated with various concentrations of HMH (0–10 mg/mL) for 2 h, followed by exposure to LPS (200 ng/mL). RT-PCR and western blotting were performed to assess the expression of iNOS and COX-2, while the intracellular NO levels were measured using NO staining. Our results showed that treatment with HMH reduced the LPS-induced transcriptional (Fig. 2A) and translational (Fig. 2B) expression of iNOS and COX-2. Additionally, HMH treatment significantly suppressed LPS-induced intracellular NO levels (Fig. 2C). The levels of NO production in cells treated with LPS alone were found to markedly higher than in untreated cells (18.5 ± 0.7 μM compared to 1.0 ± 0.1 μM), whereas treatment with HMH at concentrations of 2.5, 5, and 10 mg/mL led to reduction in NO production in a concentration-dependent manner (14.9 ± 0.8, 10.7 ± 0.4, and 5.6 ± 0.1 μM, respectively; Fig. 2D). Moreover, HMH treatment also significantly attenuated LPS-induced PGE_2_ production in a concentration-dependent manner (4688.9 ± 151.6 pg/mL in LPS alone, 4293.6 ± 151.6, 2143.1 ± 256.5, and 1342.2 ± 70.9 pg/mL at HMH concentrations of 2.5, 5, and 10 mg/mL, respectively; Fig. 2E). These findings suggest that HMH suppresses pro-inflammatory NO and PGE_2_ production in LPS-stimulated RAW 264.7 macrophages.

### 3.3 HMH reduces the production of TNF-α and IL-12 in LPS-stimulated RAW 264.7 macrophages

**Fig. 3:**
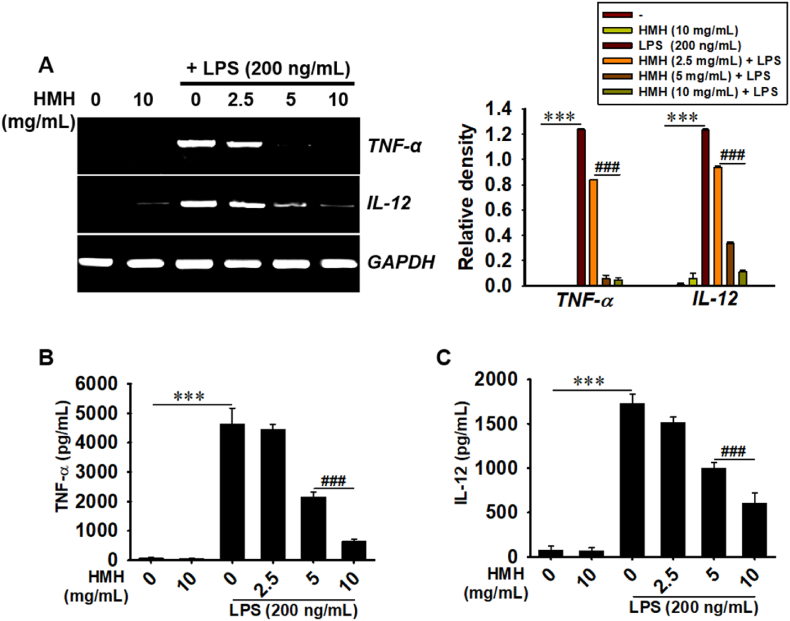
HMH reduces LPS-induced TNF-α and IL-12 secretion. RAW 264.7 macrophages (1 × 10^5^ cells/mL) were treated with 0–10 mg/mL HMH for 2 h prior to LPS (200 ng/mL) treatment. (A) Total RNA was extracted at 6 h, and RT-PCR was performed to evaluate the expression of *TNF-α* and *IL-12*. The relative expression of *TNF-α*, and *IL-12* was normalized to *GAPDH* expression. In a parallel experiment, cell-free culture media were collected at 24 h. (B) TNF-α and (C) IL-12 levels were quantified using an ELISA assay. ∗∗∗*p* < 0.001 vs. untreated cells and ^###^*p* < 0.001 vs. LPS-treated cells.

We conducted RT-PCR and ELISA to investigate the effects of HMH on the production of TNF-α and IL-12 induced by LPS. Our results showed that HMH reduced the expression of LPS-induced *TNF-α* and *IL-12* in a concentration-dependent manner (Fig. 3A). Additionally, treatment with HMH significantly decreased the levels of TNF-α from 4642.5 ± 527.8 pg/mL to 4461.2 ± 150.4, 2161.8 ± 148.1, and 643.3 ± 81.2 pg/mL at 2.5, 5, and 10 mg/mL, respectively (Fig. 3B). Similarly, HMH effectively downregulated the LPS-induced IL-12 levels from 1730.8 ± 103.7 pg/mL to 1517.6 ± 57.9, 1004.0 ± 59.0, and 605.6 ± 116.6 pg/mL at 2.5, 5, and 10 mg/mL, respectively (Fig. 3C). These findings suggest that HMH exerted significant anti-inflammatory effects by inhibiting the secretion of proinflammatory TNF-α and IL-12 in LPS-stimulated RAW 264.7 macrophages.

### 3.4 HMH inhibits LPS-triggered NF-κB activation

**Fig. 4:**
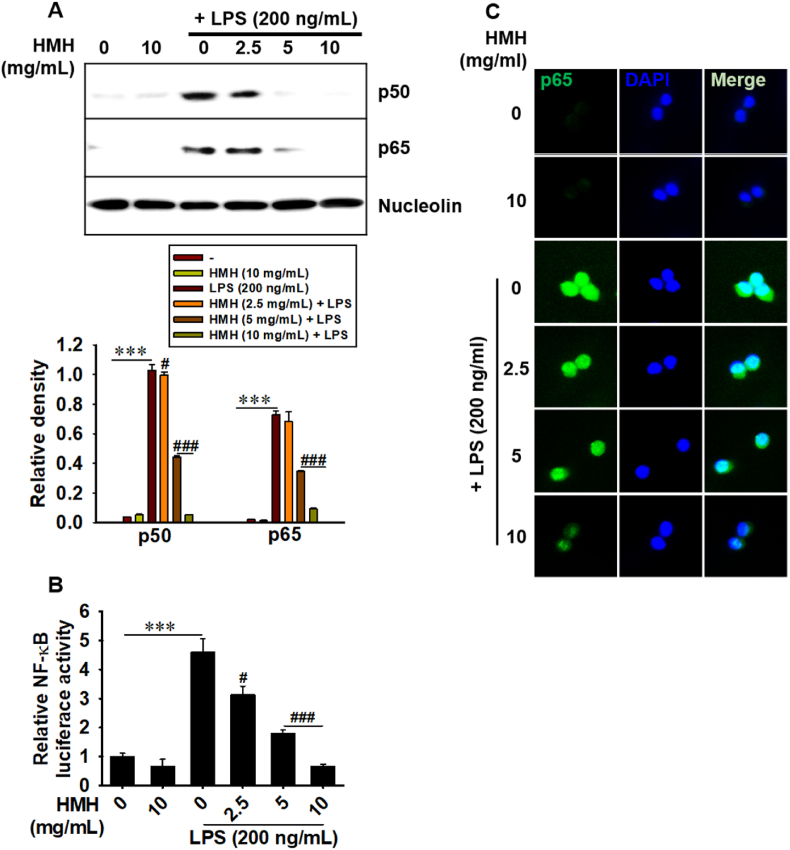
HMH inhibits LPS-triggered NF-κB activation. RAW 264.7 macrophages (1 × 10^5^ cells/mL) were treated with 0–10 mg/mL HMH for 2 h prior to LPS (200 ng/mL) treatment. (A) After 30 min of LPS exposure, nuclear proteins were extracted, and western blotting was performed to evaluate the expression of p50 and p65. Relative density of p50 and p65 was shown relative to nucleolin. (B) After 30 min of LPS treatment, NF-κB luciferase activity was measured using a GLOMAX luminometer and normalized with *Renilla* luciferase activity. (C) In a parallel experiment, cells (1 × 10^4^ cells/mL) were grown in 3 % gelatin-coated coverslips and treated with 0–10 mg/mL HMH for 2 h prior to treatment with 200 ng/mL LPS for 30 min. The cells were incubated with an anti-p65 antibody, which was captured with Alexa Fluor 488-conjugated secondary antibody. Nuclei were counterstained with 300 nM DAPI. ∗∗∗*p* < 0.001 vs. untreated cells and ^#^*p* < 0.05 and ^###^*p* < 0.001 vs. LPS-treated cells.

To determine the mechanism underlying the anti-inflammatory effects of HMH, we examined its ability to inhibit LPS-induced NF-κB activation in RAW 264.7 macrophages. Our results showed that LPS increased the nuclear accumulation of NF-κB p50 and p65 subunits, while pretreatment with HMH markedly reduced their expression in the nucleus (Fig. 4A). Furthermore, the dual-luciferase assay demonstrated that HMH markedly inhibited LPS-induced NF-κB activity (Fig. 4B). Additionally, using immunofluorescence staining, we observed that HMH concentration-dependently reduced the translocation of NF-κB p65 in the nucleus (Fig. 4C). These findings suggest that HMH suppresses LPS-induced NF-κB activation, which may contribute to its anti-inflammatory properties.

### 3.5 HMH reduces mortality, abnormality, and proinflammatory mediators in zebrafish larvae microinjected with LPS

**Fig. 5:**
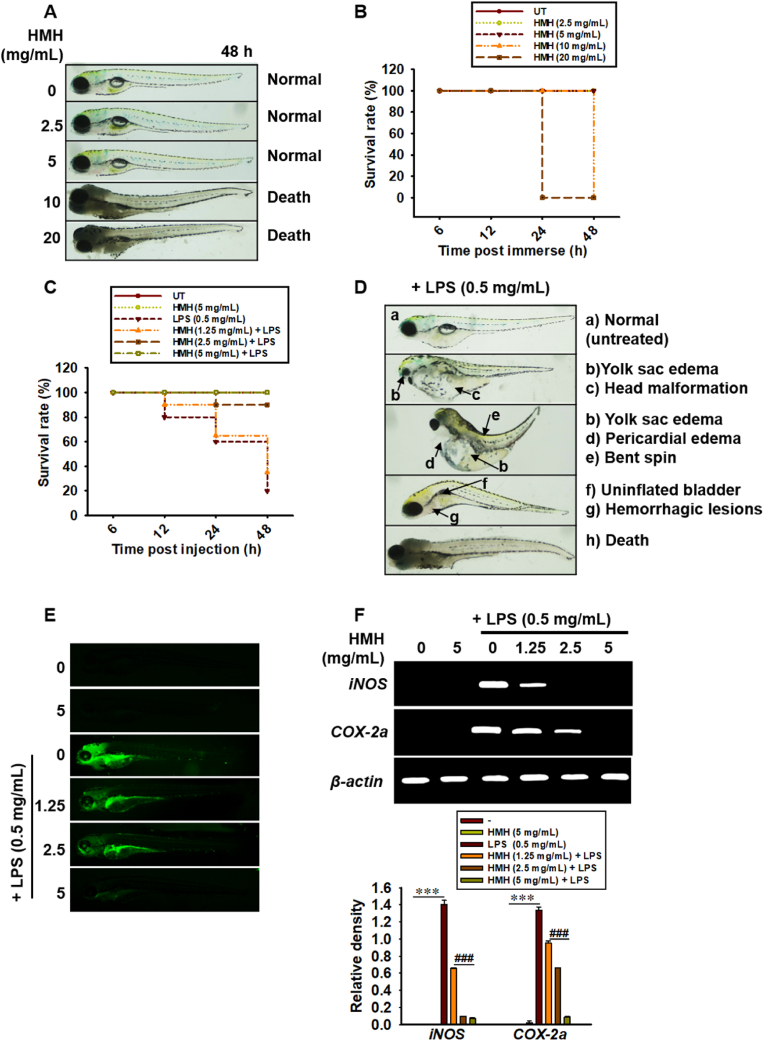
HMH attenuates mortality, abnormalities, and proinflammatory mediator expression in LPS-microinjected zebrafish larvae. (A) Representative image of 3 dpf larvae (n = 10 in each group, triplicated) exposed to 0–20 mg/mL HMH for 48 h. (B) The survival rate of 3 dpf zebrafish larvae with 0–5 mg/mL HMH treatment. Zebrafish larvae at 3 dpf were microinjected with LPS (0.5 mg/mL, 2 nL per larva) and treated with 0–5 mg/mL HMH. (C) The survival rate of zebrafish larvae was determined under a stereomicroscope. (D) Representative images of LPS-microinjected zebrafish larvae. (E) Zebrafish larvae were stained with 10 μM DAF-FMDA for 10 min, and the images were captured using a CELENA S Digital Imaging System. (F) Total RNA was extracted at 18 h, and RT-PCR was performed to evaluate the expression of *iNOS* and *COX-2a*. The relative density of *iNOS* and *COX-2a* expression was shown relative to *β-actin*. ∗∗∗*p* < 0.001 vs. untreated zebrafish larvae and ^###^*p* < 0.001 vs. LPS-microinjected zebrafish larvae.

To evaluate the potential toxicity of HMH in vivo, zebrafish larvae were exposed to various concentrations of HMH (0–20 mg/mL) for 48 h at 3 dpf. At 20 mg/mL, HMH caused 100 % mortality at 24 h, whereas at 10 mg/mL, 100 % mortality was observed after 48 h (Fig. 5A and 5B). To evaluate the effectiveness of HMH in preventing mortality and abnormalities in an LPS-microinjected zebrafish endotoxic shock model, zebrafish larvae at 3 dpf were microinjected with LPS (0.5 mg/mL, 2 nL in each larva) and treated with non-toxic concentrations of HMH. As shown in Fig. 5C, approximately 80 % of the zebrafish larvae that were microinjected with LPS died after 48 h, and the remaining larvae exhibited severe morphological abnormalities, including head malformation, bent spine, yolk sac edema, pericardial edema, uninflated bladder, and hemorrhagic lesions (Fig. 5D). However, treatment with 5 mg/mL HMH completely inhibited the LPS-induced mortality and abnormalities in zebrafish larvae (Fig. 5C). Additionally, HMH treatment reduced the LPS-induced levels of intracellular NO (Fig. 5E). Furthermore, HMH decreased the LPS-induced expression of pro-inflammatory genes, such as *iNOS* and *COX-2a*, in a concentration-dependent manner (Fig. 5F). These results suggest that HMH has the potential to inhibit mortality and abnormalities caused by LPS in zebrafish larvae, as well as to suppress pro-inflammatory genes.

### 3.6 HMH reduces the depolarization of ΔΨm and mtROS production in LPS-stimulated RAW264.7 macrophages and zebrafish larvae

**Fig. 6:**
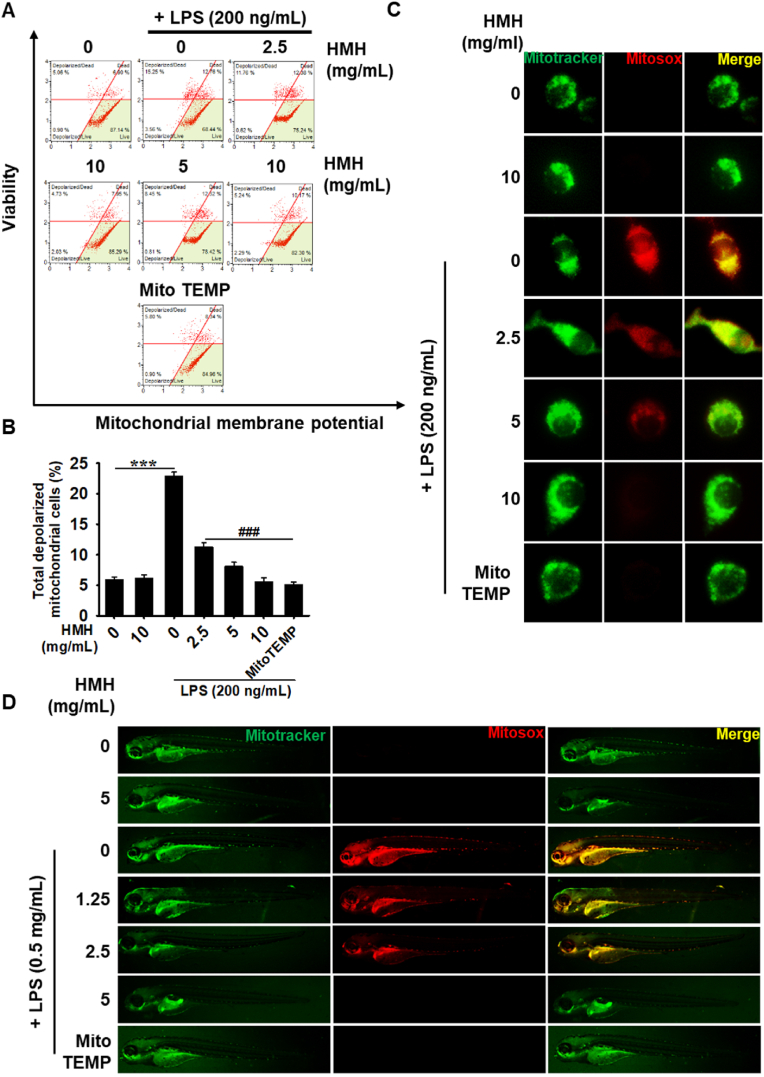
HMH reduces the mitochondrial membrane potential (ΔΨm) depolarization and mtROS production. RAW 264.7 macrophages (1 × 10^5^ cells/mL) were treated with 0–10 mg/mL HMH for 2 h prior to LPS (200 ng/mL) treatment for 24 h. (A) Cells were harvested and stained using a Muse Mitopotential Kit, and the ΔΨm was measured using flow cytometry. (B) Total ΔΨm depolarized cells (%) were shown. (C) In a parallel experiment, cells were stained using 5 μM MitoTracker Green for 30 min and subsequently with 10 μM MitoSOX Red for 10 min in the presence or absence of 5 μM MitoTEMPO. Fluorescence images were captured using a CELENA S Digital Imaging System. (D) The 3 dpf larvae were microinjected with LPS (0.5 mg/mL, 2 nL per larva) and treated with 0–5 mg/mL HMH for 24 h. Larvae were stained using 10 μM MitoSOX Red for 30 min and 5 μM MitoTracker Green for 10 min in embryo medium in the presence or absence of 10 μM MitoTEMPO, and the images were captured using a CELENA S Digital Imaging System. ∗∗∗*p* < 0.001 vs. untreated cells and ^###^*p* < 0.001 vs. LPS-treated cells.

We investigated the effects of HMH on ΔΨm and mtROS production in LPS-stimulated RAW 264.7 macrophages. Our findings showed that LPS significantly increased the population of cells with depolarized ΔΨm from 5.9 ± 0.4 to 22.9 ± 0.6, whereas HMH treatment reduced the populations in a concentration-dependent manner (11.3 ± 0.7, 8.1 ± 0.7, and 5.6 ± 0.6 at 2.5, 5 and 10 mg/mL, respectively; Fig. 6A and B). Immunofluorescence staining showed that LPS induced the upregulation of mtROS in the mitochondria, but the highest concentration of HMH (10 mg/mL) completely suppressed LPS-induced mtROS production similar to MitoTEMPO, an inhibitor of mtROS production. Additionally, LPS microinjection induced mtROS production in zebrafish larvae, whereas HMH treatment significantly decreased mtROS production in a concentration-dependent manner (Fig. 6D). We also observed that MitoTEMPO suppressed LPS-microinjected mtROS production in zebrafish larvae and that its inhibitory effect was comparable to that of 5 mg/mL HMH. These findings suggest that HMH stabilizes ΔΨm against LPS and reduces excessive mtROS production in RAW264.7 macrophages and zebrafish larvae.

### 3.7 HMH protects against oxidative damage induced by LPS stimulation by activating the Nrf2/HO-1 pathway

**Fig. 7:**
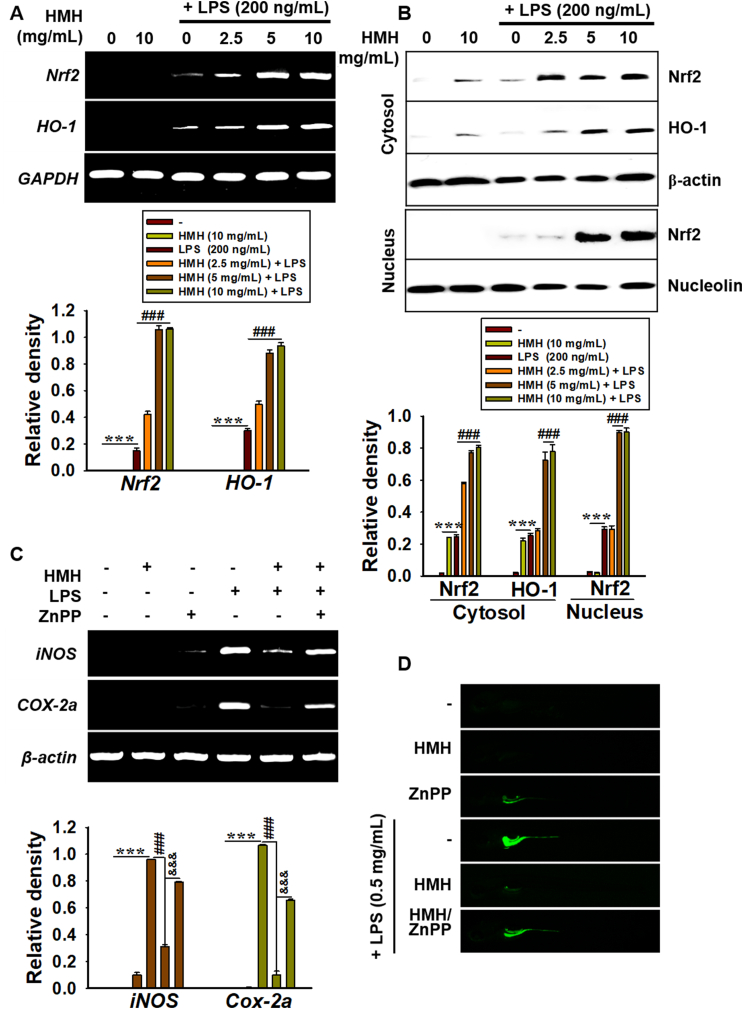
HMH protects against LPS-induced oxidative stress via the Nrf2/HO-1 pathway. RAW264.7 macrophages (1 × 10^5^ cells/mL) were treated with 0–10 mg/mL HMH for 2 h prior to LPS (200 ng/mL) treatment for 24 h. (A) Total RNA was extracted at 6 h, and RT-PCR was performed to evaluate *Nrf2* and *HO-1* expression. (B) In a parallel experiment, nuclear and cytosolic proteins were extracted at 24 h, and western blotting was performed to evaluate Nrf2 and HO-1 expression. The relative density of Nrf2 and HO-1 expression was shown relative to each control. (C) Zebrafish larvae at 3 dpf were microinjected with LPS (0.5 mg/mL, 2 nL per larva) and treated with HMH (5 mg/mL) or ZnPP (10 μM) for 18 h. Total RNA was extracted, and RT-PCR was performed to evaluate *iNOS* and *COX-2a* expression. The relative density of *iNOS* and *COX-2a* expression was shown relative to *β-actin*. (D) In a parallel experiment, zebrafish larvae were stained using 5 μM DAF-FMDA, and the images were captured using a CELENA S Digital System. ∗∗∗*p* < 0.001 vs untreated group, ^###^*p* < 0.001 vs LPS-treated group, and ^&&&^*p* < 0.001 vs. LPS/HMH-treated group.

To determine whether HMH protects against LPS-induced oxidative damage by activating the Nrf2/HO-1 signaling pathway, we examined the expression of Nrf2 and HO-1. Our results showed that HMM increased Nrf2 and HO-1 expression in a concentration-dependent manner at both the transcriptional (Fig. 7A) and translational (Fig. 7B) levels. Furthermore, Western blot analysis revealed that HMH significantly increased nuclear Nrf2 levels in the presence of LPS. To further investigate the anti-inflammatory effects of HMH in zebrafish larvae, we used ZnPP, a potent competitive inhibitor of HO-1, to suppress HO-1 activity. Zebrafish larvae at 3 dpf were pretreated with ZnPP for 2 h, followed by exposure to LPS and HMH, and proinflammatory gene expression and NO levels were measured. As shown in Fig. 7C, LPS significantly increased *iNOS* and *COX-2a* expression irrespective of ZnPP treatment. In addition, the inhibitory effect of HMH on LPS-induced gene expression was significantly attenuated by ZnPP treatment. Furthermore, fluorescence staining of intracellular NO showed that ZnPP alone caused a slight increase in NO production compared to that in untreated zebrafish larvae, which was further increased in the presence of LPS. Importantly, the inhibitory effect of HMH on LPS-induced NO production was abolished by ZnPP, indicating that HO-1 is a crucial regulator of HMH-mediated NO inhibition in zebrafish larvae. Overall, these findings indicate that HMH protects against LPS-induced inflammatory response by activating the Nrf2-HO-1 pathway.

## 4 Discussion

Many scientific studies have explored the health benefits of honey, which contains essential bioactive compounds, such as polyphenols, flavonoids, and vitamins, that have antioxidant, anti-inflammatory, and anti-carcinogenic effects.^24^^,^^25^ However, owing to the variation in compounds from different flowers in each season, standardizing the beneficial effects of multifloral honey remains a challenge. Studies on monofloral honey can provide insights into how honey functions in medicinal therapeutics.^20^ A recent study by Park et al. demonstrated that HMH contains high concentrations of phenolic compounds, flavonoids, and minerals with strong antioxidant and antimicrobial properties in vitro.^21^ Furthermore, Mei-Chen Li et al. observed five distinct classes of flavonoids in the phytochemical study and Yonghwan Son et al. performed a UPLC methodology to distinguish four biologically active compounds, including ampelopsin, taxifolin, myricetin, and quercetin. Additionally, HMH inhibited *Enterococcus faecalis*-induced IL-8 expression in HT-29 gastrointestinal epithelial cells.^22^ However, whether HMH reduces inflammation during endotoxemia remains unclear. In this study, we found that HMH inhibited LPS-triggered inflammation and endotoxemia by suppressing the NF-κB signaling pathway and mtROS production, suggesting potential health benefits for inflammatory responses. These findings emphasize the importance of investigating the effects of monofloral honey on various health conditions and its underlying mechanisms for the development of new therapeutic interventions.

The transcription factor NF-κB plays a crucial role in regulating immune and inflammatory responses by controlling the expression of genes involved in these processes.^26^ The inactivation of NF-κB can increase susceptibility to pathogen infections. Endotoxemia, which is characterized by the presence of LPS in the bloodstream, can cause organ failure.^1^^,^^2^ During endotoxemia, immune cells recognize LPS through the Toll-like receptor 4 (TLR4)/MD2 complex, resulting in the activation of the NF-κB signaling pathway and the production of proinflammatory cytokines and free radicals, such as ROS and RNS.^9^ Given its involvement in the regulation of cytokines, chemokines, and free radicals, NF-κB plays a crucial role in mediating immune and inflammatory responses to endotoxemia.^27^, ^28^, ^29^ The results of this study suggest that HMH may exhibit potential therapeutic benefits against endotoxemia by inhibiting the NF-κB signaling pathway. Further research into the bioactive compounds present in HMH may lead to the development of novel therapeutic strategies for immune and inflammatory disorders.

The activation of the transcription factor Nrf2 activation plays a crucial role in reducing inflammation and oxidative stress in animal models of endotoxemia.^30^ Studies have also suggested that Nrf2 activation may improve the survival of animals with endotoxemia by increasing the expression of genes involved in antioxidant defense and cellular detoxification, including HO-1.^14^^,^^31^^,^^32^ Moreover, Nrf2 activation may modulate the inflammatory response to endotoxemia by inhibiting NF-κB activity and reducing the expression of proinflammatory cytokines.^33^^,^^34^ Although the relationship between Nrf2 and endotoxemia is complex and requires further investigation, promising evidence suggests that Nrf2 activation may be a useful therapeutic strategy for the prevention or treatment of endotoxemia. In this study, we found that HMH exerts an anti-endotoxemic effect by promoting Nrf2-mediated HO-1 activation. The proinflammatory mediators and free radicals induced by LPS were reversed by ZnPP, indicating that the activation of the Nrf2/HO-1 pathway is responsible for the anti-inflammatory and antioxidant effects of HMH in response to LPS stimulation. However, the role of HO-1 in the pathophysiology of sepsis remains nuclear. Although many previous studies have demonstrated that HO-1 inhibits endotoxemia and sepsis, it also plays a role in organ damage.^35^ Therefore, further studies are required to elucidate the role of HO-1 in endotoxemia and sepsis.

In summary, this study has provided valuable insights into the potential health benefit of HMH, which demonstrated significant anti-inflammatory and anti-endotoxemia effects against LPS-induced responses in RAW 264.7 macrophages and zebrafish larvae. The observed increase in Nrf2-mediated HO-1 expression by HMH suggests that its anti-inflammatory and anti-endotoxemia effects are mediated by inhibiting the NF-κB signaling cascade. These findings highlighted the potential of HMH as a therapeutic agent for endotoxemia and related inflammatory disorders. However, further studies are required to optimize the concentration of HMH and investigate its long-term safety and efficacy in preclinical models of LPS-induced endotoxemia. Overall, the results presented in this study provide compelling evidence for the development of novel therapies using HMH for the treatment of endotoxemia and related inflammatory conditions.

## Declaration of competing interest

The authors declare that they have no known competing financial interests or personal relationships that could have appeared to influence the work reported in this paper.
